# Analysis of a Modern Hybrid and an Ancient Sugarcane Implicates a Complex Interplay of Factors in Affecting Recalcitrance to Cellulosic Ethanol Production

**DOI:** 10.1371/journal.pone.0134964

**Published:** 2015-08-07

**Authors:** Viviane Guzzo de Carli Poelking, Andrea Giordano, Maria Esther Ricci-Silva, Thomas Christopher Rhys Williams, Diego Alves Peçanha, Marília Contin Ventrella, Jorge Rencoret, John Ralph, Márcio Henrique Pereira Barbosa, Marcelo Loureiro

**Affiliations:** 1 Departamento de Biologia Vegetal, Universidade Federal de Viçosa, Viçosa, Minas Gerais, Brazil; 2 Biochemistry Department, and the Great Lakes Bioenergy Research Center, University of Wisconsin, Madison, Wisconsin, United States of America; 3 Departamento de Fitotecnia, Universidade Federal de Viçosa, Viçosa, Minas Gerais, Brazil; National Renewable Energy Lab, UNITED STATES

## Abstract

Abundant evidence exists to support a role for lignin as an important element in biomass recalcitrance. However, several independent studies have also shown that factors apart from lignin are also relevant and overall, the relative importance of different recalcitrance traits remains in dispute. In this study we used two genetically distant sugarcane genotypes, and performed a correlational study with the variation in anatomical parameters, cell wall composition, and recalcitrance factors between these genotypes. In addition we also tracked alterations in these characteristics in internodes at different stages of development. Significant differences in the development of the culm between the genotypes were associated with clear differential distributions of lignin content and composition that were not correlated with saccharification and fermentation yield. Given the strong influence of the environment on lignin content and composition, we hypothesized that sampling within a single plant could allow us to more easily interpret recalcitrance and changes in lignin biosynthesis than analysing variations between different genotypes with extensive changes in plant morphology and culm anatomy. The syringyl/guaiacyl (S/G) ratio was higher in the oldest internode of the modern genotype, but S/G ratio was not correlated with enzymatic hydrolysis yield nor fermentation efficiency. Curiously we observed a strong positive correlation between ferulate ester level and cellulose conversion efficiency. Together, these data support the hypothesis that biomass enzymatic hydrolysis recalcitrance is governed by a quantitative heritage rather than a single trait.

## Introduction

The plant cell wall is considered to be the most abundant renewable energy resource on Earth and is a promising substrate for second generation biofuel production [1]. Ethanol production from lignocellulosic biomass involves four stages: pretreatment, saccharification (enzymatic hydrolysis), fermentation, and product separation/purification [2]. Currently, the required pretreatment and the enzymatic hydrolysis steps remain extremely costly due to cell wall recalcitrance [3].

Plant cell walls are resistant to chemical, physical, and microbial degradation all of which are determined in large part by the cell wall components and their interactions [4,5]. Lignin content and composition were reported as principal factors limiting the conversion of cell wall polysaccharides to sugars and hence limiting biofuel production [6–14]. Other factors in grasses such as polysaccharide cross-linking (including that mediated by ferulate), lignin-carbohydrate linkages (including those mediated by ferulate and diferulates), cellulose crystallinity, lignin/hemicellulose structures, and biomass porosity, are reported to contribute to cell wall recalcitrance [15,16].

Despite several studies reporting that the presence of xylans has a negative effect on cellulose conversion [17,18], a recent report showed that xylans can improve enzymatic digestibility [19]. Other cell wall components such as acetyl groups and pectins were also suggested to restrict cellulose accessibility [20–23].

In addition to cellulose, hemicelluloses, and lignin, grass (monocot) cell walls contain high levels of hydroxycinnamates, particularly ferulate and *p*-coumarate [24]. *p*-Coumarate is found acylating hemicelluloses and lignin, whereas ferulate almost entirely acylates just hemicelluloses [25]. Both hydroxycinnamates, but primarily ferulate, are also important in cross-linking these two components, hemicelluloses and lignins, within the cell wall [25]. In the cell walls of most grasses, *p*-coumarate primarily acylates S lignin monomers and its accumulation can be correlated with lignin deposition [26].

Hydroxycinnamates were extensively studied as a negative factor in cell wall digestibility [27–29], whereas their effects on enzymatic hydrolysis and fermentation are relatively poorly understood. Ester- and ether-linked phenolics with lignin and other cell wall polymers are suggested to act as a barrier impeding enzyme access to cellulose [30–33]. Etherified ferulate was negatively correlated with cell wall digestibility reinforcing the likely negative effect of ferulate cross-linkages on cell wall digestibility [27,32–35]. In addition, ferulic acid has been shown to have an inhibitory effect on yeast, requiring additional treatments to maintain ethanol production [36]. *p*-Coumarate esters were reported as being negatively correlated with cell wall digestibility in several studies [29,37,38].

Recent studies on biofuel feedstocks such as Miscanthus [28], sugarcane [39], and maize [27] showed that the high diversity of cell wall composition and structure affects biomass recalcitrance. Characterization of germplasm resources therefore enables us to select and breed varieties with high ethanol productivity and low recalcitrance.

Sugarcane bagasse is a promising substrate for bioethanol production because of its high cellulose content (50%) that could be converted into glucose and almost completely fermented [40]. Plant breeding between species of the genus *Saccharum* (*S*. *spontaneum*, *S*. *robustum*, *S*. *officinarum*, *S*. *barberi*, *S*. *sinense* and *S*. *edule*) has resulted in hybrids that gave the genetic background of the cultivars that can be found today. The genotype RB867515, henceforth just denoted as ‘RB’, is a highly productive modern hybrid and the most heavily planted in Brazil [41].

In this study we address the effects of cell wall composition through culm maturity that contribute to recalcitrance in the modern hybrid (RB) and the ancient genotype *S*. *spontaneum* (henceforth denoted as AG). Today’s hybrids contain between 15–27.5% of this parental ancient genotype genome [42]. We studied anatomical features, the deposition pattern of the main cell wall components, the phenolic compound profile, and the saccharification and fermentation efficiency of AG and the modern hybrid RB throughout culm development. The results are expected to assist sugarcane breeding programs aimed at optimizing bioethanol production from sugarcane.

## Results

### Anatomical differences between ancient and modern sugarcane genotypes

Anatomical features of the intermediate internode (IN5) from AG and genotype RB differed significantly (Table 1). The modern hybrid RB showed a higher percentage of vascular bundles (25%), total fiber (41%), non-lignified fiber (160%), parenchymal cell diameter, metaxylem cell wall thickness, and metaxylem vessel diameter. AG had higher lignified fiber percentage (38%), fiber length (65%), fiber wall thickness (88%), and lumen diameter (36%).

**Table 1:** Micromorphometric analysis of internode 5 from cultivar RB867515 (RB) and *S*. *spontaneum* (AG).

Morphological Characteristics	RB8675115 (RB)		*S*. *spontaneum* (AG)		t value
	Mean	SE	Mean	SE	p *<0*.*05*
% vascular bundles	19.82*	0.65	15.70	0.52	0.00
Total fibers (%)	25.04*	2.70	17.70	1.04	0.04
Non-lignified fibers (%)	16.94*	2.48	6.51	1.69	0.01
Lignified fibers (%)	8.10	0.50	11.20*	0.68	0.00
Fiber diameter (μm)	7.31	0.17	11.40*	0.22	0.00
Fiber length (μm)	738.30	54.70	1,224*	37.21	0.00
Fiber wall thickness (μm)	1.38	0.04	2.60*	0.09	0.00
Lumen diameter (μm)	4.53	0.24	6.10*8	0.35	0.00
Thickness of metaxylem vessel walls (μm)	9.15*	1.32	5.20	0.26	0.02
Metaxylem vessels diameter (μm)	57.30	0.61	61.04*	0.24	0.00
Parenchyma cells diameter (μm)	86.38*	1.88	77.90	1.70	0.00

Values are mean and standard error (n = 5). Asterisks indicate a significant difference between the genotypes (t-test, p<0.05).

### Analysis of cell wall composition reveals differences throughout development and between genotypes

**Fig 1:**
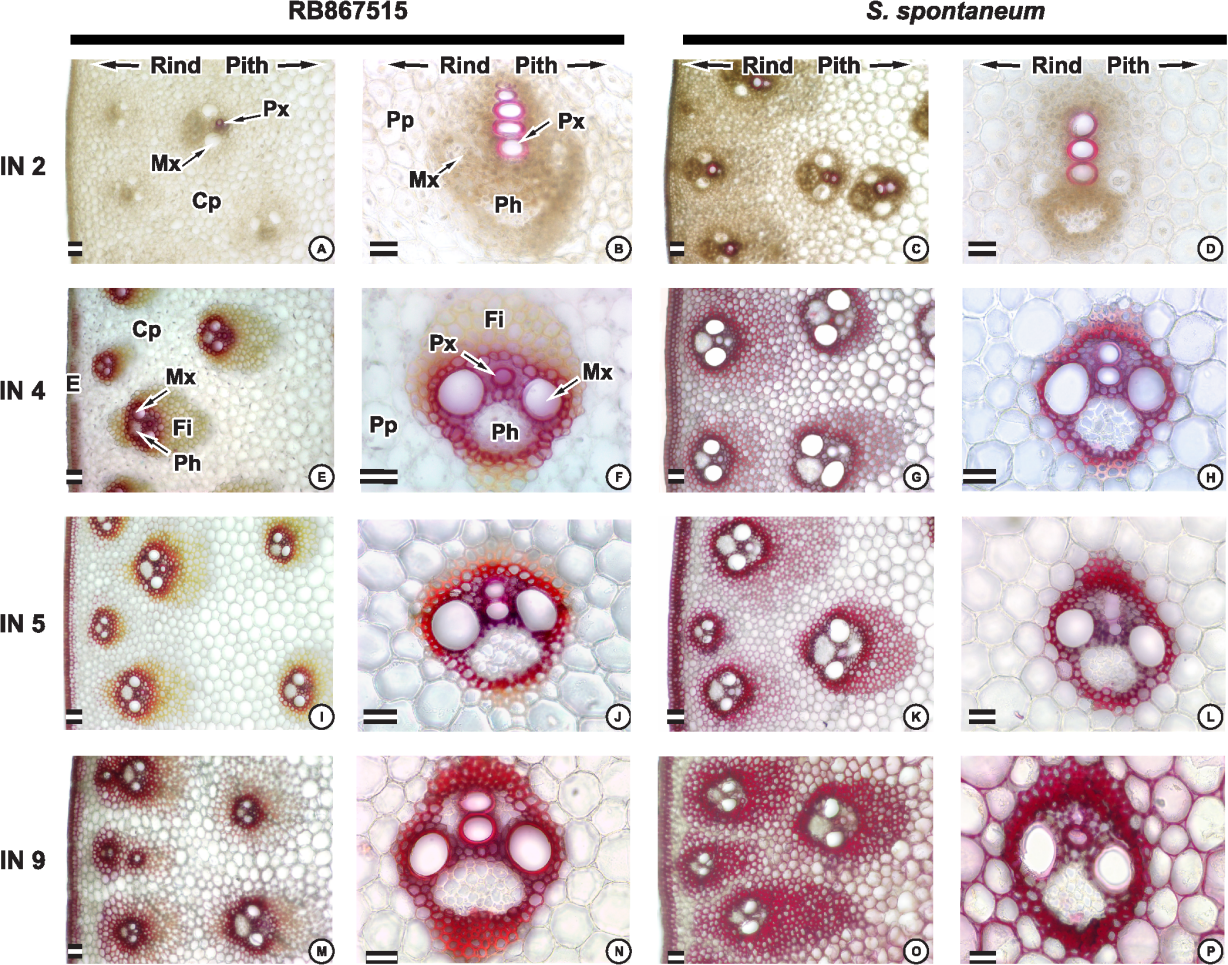
Wiesner staining (phloroglucinol-HCl) of transverse sections of internodes in distantly related sugarcane genotypes. Transverse sections of-cultivar RB867515 and *S*. *spontaneum*. Second internode (A, B and C, D), 4^th^ internode (E, F and G, H), 5^th^ internode (I, J and K, L) and 9^th^ internode (M, N and O, P). Detail of the vascular bundle of the central region of the second internode(B, D), 4^th^ internode (F, H), 5^th^ internode (J,L), 9^th^ internode (N, P). Photographs are representative of at least six samples. The labels “Rind” and “pith” indicate their positions in the sections. E: epidermal cells, Fi: fiber, Ph: phloem, CP: cortical parenchyma, Pp: pith parenchyma, Mx: metaxylem, Px: protoxylem. Scale bar = 50 μm.

**Fig 2:**
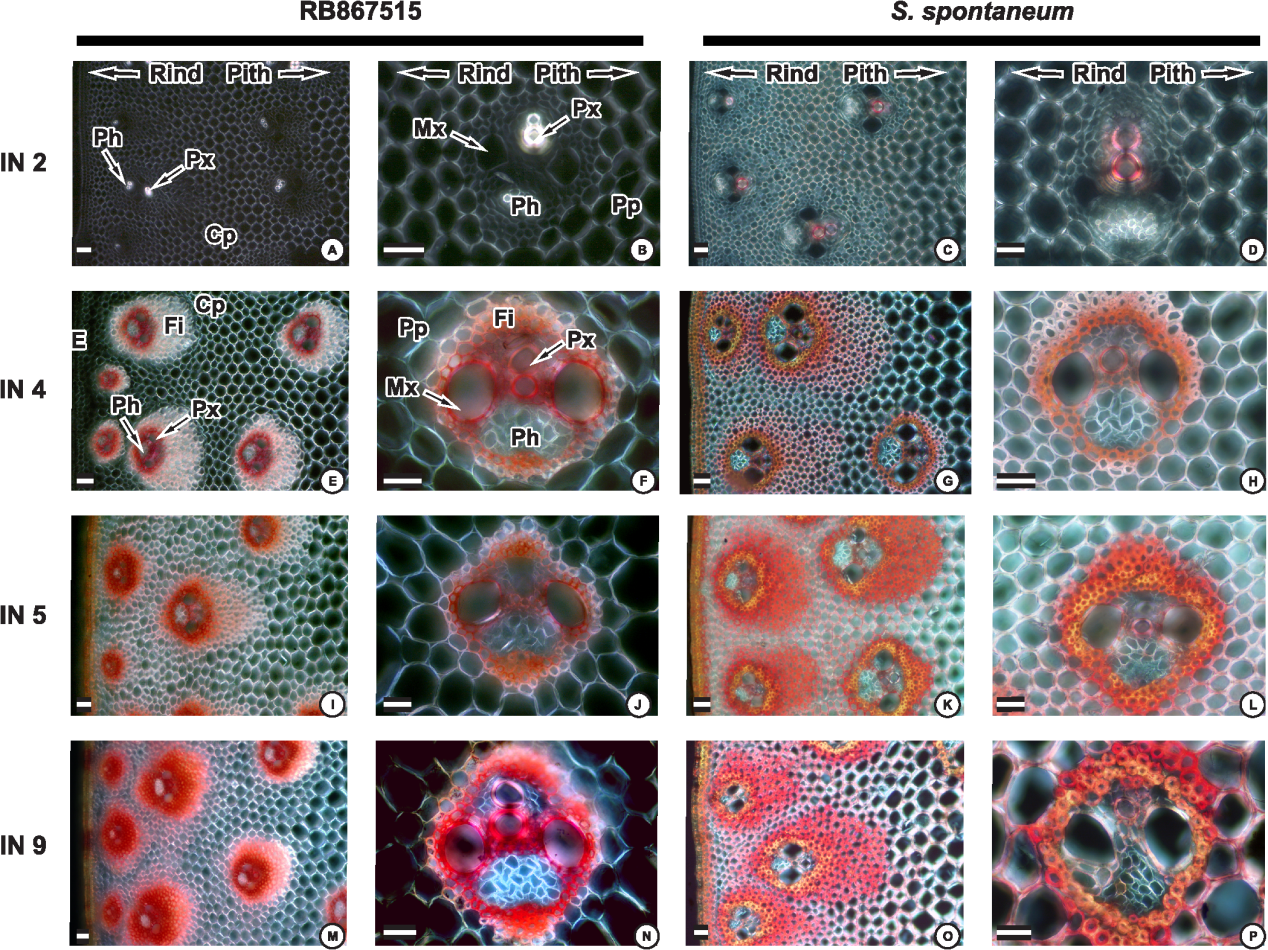
Maüle staining of internode transverse sections observed by light microscopy using cross-polarizing optics. Transverse sections of cultivar RB867515 and *S*. *spontaneum* Second internode (A, B and C, D), 4^th^ internode (E, F and G, H), 5^th^ internode (I, J and K, L) and 9^th^ internode (M, N and O, P). Red staining indicates the presence of syringyl residues in the plant secondary cell wall. Detail of the vascular bundle of the central region of the second internode (B, D), 4^th^ internode (F, H), 5^th^ internode (J,L), 9^th^ internode (N, P). Photographs are representative of at least six samples. Rind and pith indicate their positions in the sections. E: epidermal cells, Fi: fiber, Ph: phloem, CP: cortical parenchyma, Pp: pith parenchyma, Mx: metaxylem, Px: protoxylem. Scale bar = 50 μm.

Lignin deposition in young (IN2), intermediate (IN5), and mature (IN9) internodes from the genotype RB and AG were stained using phloroglucinol-HCl (Wiesner staining) (Fig 1) and Mäule staining (Fig 2).

Histochemical staining clearly showed that the ancient genotype AG has greater lignification in fiber and parenchyma cells in IN5 and IN9, but no clear differences were observed in vascular bundle lignification.

Wiesner staining, which reacts with cinnamaldehyde end-groups in both G and S lignin, showed a differential lignification pattern in the intermediate and mature internodes. In the intermediate internodes of RB (IN4 and IN5), lignification is restricted to protoxylem, metaxylem, and the first layers of fibers surrounding the vascular bundles and epidermis (Fig 1E, 1F, 1I and 1J) whereas in AG lignification extends to parenchyma layers, several fiber layers surrounding vascular vessels, and cortical parenchyma (Fig 1G,1 H, 1K and 1L).

In the mature internode (IN9) AG lignin deposition was extended to the cortical area and several layers associated with cortical vascular bundles, cortical parenchyma and pith (Fig 1O and 1P) whereas only a few layers surrounding vascular vessels of the cortical area were stained in RB (Fig 1M and 1N),

Syringyl units were detected using Mäule reagent. In the youngest internode S lignin was only detected in vascular tissue of *S*. *spontaneum* suggesting an earlier deposition of S lignin (Fig 2C and 2D). Cross sections of the intermediate internodes (IN4 and IN5) were stained similarly. Deposition was observed in the metaxylem tissues, fibers surrounding vascular bundles and the first parenchyma cell layers (Fig 2E–2H and 2I–2L).

S-lignin deposition in IN9 of RB was evenly spread within the fibers nearest to the vascular tissues, mainly in the cortical region (Fig 2M and 2N). However, the pattern of S-lignin deposition in IN9 of AG (Fig 2O and 2P) was similar to that observed in IN5, with the exception of the fibers next to the vascular tissue (not detected).

**Fig 3:**
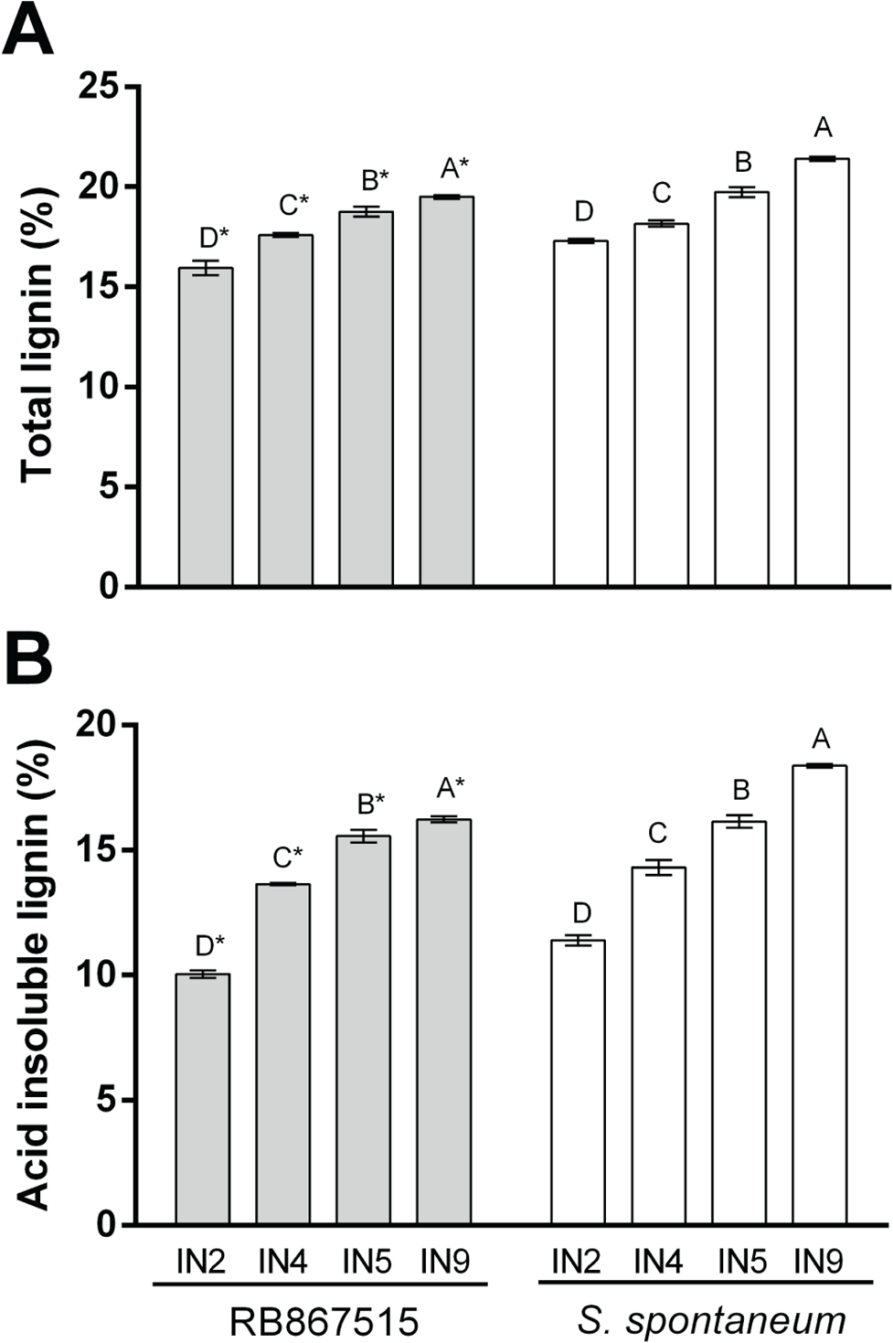
Lignin content of dry cell wall from RB867515 (RB) and *S*. *Spontaneum* (AG) internodes throughout development. (A) total lignin (%) in internodes (IN2, IN4, IN5, IN9) from RB867515 (grey bars) and *S*. *spontaneum* (white bars). (B) acid insoluble lignin (%) in internodes (IN2, IN4, IN5, IN9) from RB867515 (grey bars) and *S*. *spontaneum* (white bars). Bars with common letters were not significantly different (Tukey test, p<0.05). Asterisks indicate a significant difference (t-test, p<0.05) between same internode. Error bars indicate SE of technical replicates of a pool of biological samples (n = 3).

**Fig 4:**
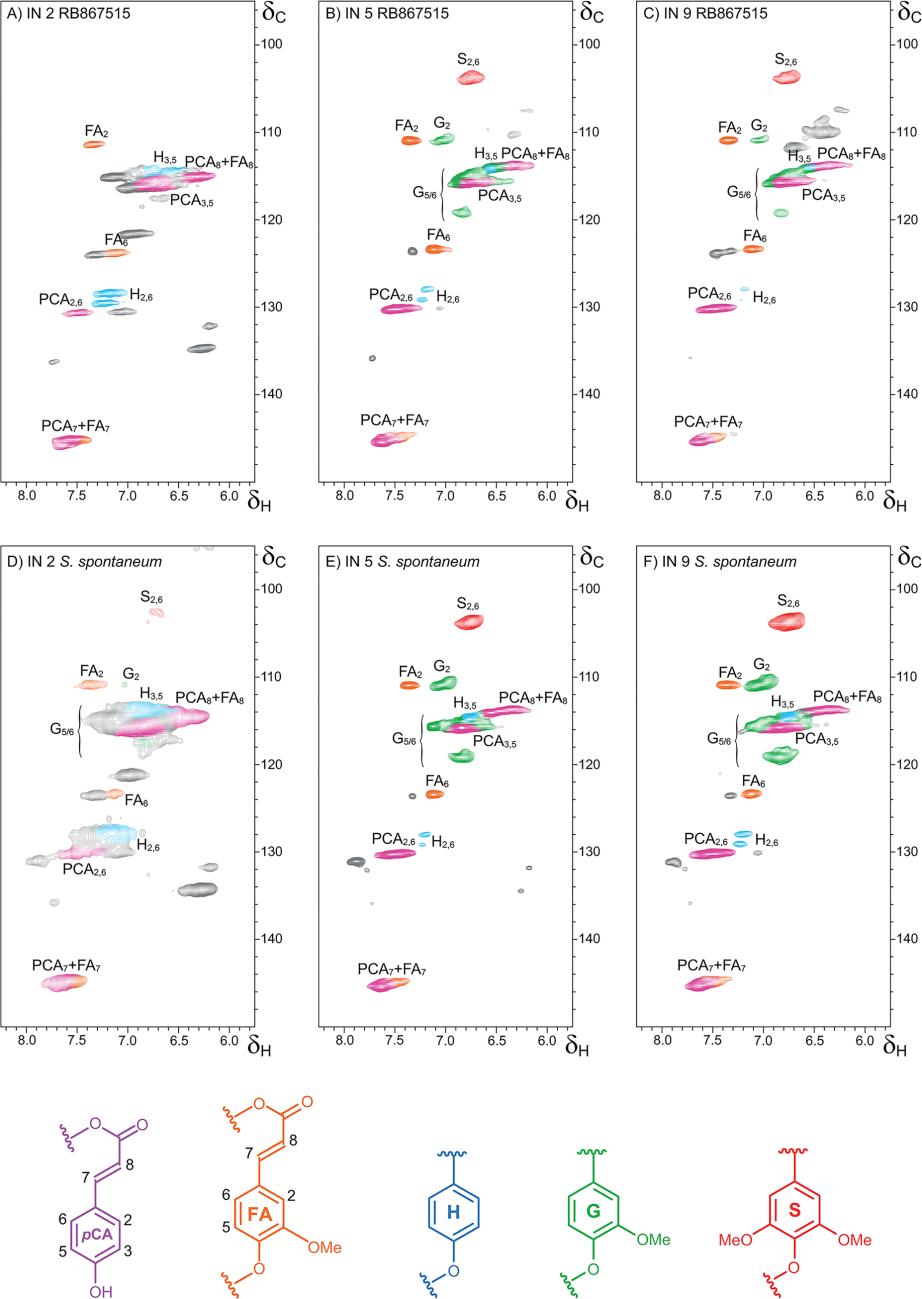
2D NMR spectra revealing lignin unit composition. **Partial short-range** ^**1**^ **H–** ^**13**^ **C(HSQC) correlation spectra (aromatic regions).** A) RB867515 (RB) internode 2, B) RB internode 5, C) RB internode 9, D) *S*. *spontaneum* (AG) internode 2, E) AG internode 5, F) AG internode 9. S = syringyl, G = guaiacyl, H = *p*-hydroxyphenyl, *P*CA = *p-*coumarate, FA = ferulate.

Cell wall composition was analyzed during stem development in young, intermediate and mature internodes of both genotypes. Despite very clear differences in anatomical and histochemical patterns of lignin and syringyl deposition in different culm tissues, there was a minor but significant difference in lignin content between genotypes. Considering all internodes, on average the lignin content was around 10% higher in the ancient genotype (AG). As expected, total and acid-insoluble (Klason) lignin deposition increased significantly with internode maturation in both genotypes (Fig 3A and 3B, S1 Table). Acid-insoluble lignin content was also significantly higher in *S*. *spontaneum* in all internodes. NMR spectroscopy was used to determine the molar percentages of lignin aromatic units and hydroxycinnamates (Fig 4, Table 2) in cell wall gel samples. Syringyl units were more abundant in lignin from RB in all internodes compared to AG, whereas guaiacyl units were more abundant in internodes 5 and 9 of AG compared to the same internodes in RB. This difference in relative abundance of S and G units led to a greater S/G ratio in all internodes in RB when compared to the same internode in AG. H units represented a greater percentage of lignin in internodes 5 and 9 of RB; in internode 2 the relative abundance of H was greater in AG, though this measurement is compromised by the presence of proteins in the sample. The two genotypes showed a similar pattern of change in molar percentage with internode maturity; in both cases S and G abundance increased from internode 2 to internode 5 whilst H abundance decreased between internodes 2 and 9 and S/G ratio also increased with increasing internode maturity.

**Table 2:** Aromatic units, *p*-coumarates and ferulates from Integration of ^1^H–^13^C correlation signals in the HSQC spectra of the GEL sample.

	RB867515 (RB)			*S*.*spontaneum* (AG)		
	IN2	IN5	IN9	IN2	IN5	IN9
Lignin aromatic units^a^						
H (%)	64.5^b^	13.1	7.6	74.8^b^	7.0	6.3
G (%)	22.0	46.9	39.4	16.3	59.0	55.6
S (%)	13.5	40.0	52.9	8.9	34.1	38.1
S/G ratio	0.6	0.9	1.3	0.5	0.6	0.7
*p*-Hydroxycinnamates^c^						
*p*-Coumarate (%)	27.6	91.0	109.9	20.9	65.3	49.7
Ferulate (%)	47.7	71.8	65.4	33.6	33.9	22.4
Ferulate/*p*-coumarate ratio	1.7	0.8	0.6	1.6	0.5	0.5
S/Ferulates ratio	0.3	0.6	0.8	0.3	1.0	1.7
S/*p*-Coumarate ratio	0.5	0.4	0.5	0.4	0.5	0.8

^a^Molar percentages (H+G+S = 100)

^b^Overestimated due to the presence of proteins

^c^Molar content as percentage of lignin content (H+G+S = 100)

Chemical analysis gave similar results to those obtained using NMR spectroscopy (S1 Table). S/G ratio increased with internode maturity in both genotypes, and a significantly greater S/G ratio was detected in IN9 of RB compared to the same internode in AG. In addition G units were more abundant than S units in all internodes and the abundance of S and G tended to increase with internode maturity. Quantitative differences between the results likely reflect the different techniques used and the fact that the NMR method does not require the prior fractionation of samples.

#### Changes in phenolics and other major cell wall compounds throughout development in distantly related sugarcane genotypes

NMR analysis indicated that the abundance of *p*-coumarate (PCA) in relation to lignin units increased between internodes 2 and 5 in both genotypes and was greater in RB in all internodes. Ferulate as a percentage of lignin content was affected to a lesser extent by internode maturity than PCA. Additionally, this component responded in a different manner between the genotypes; the abundance of PCA was higher in internodes 5 and 9 of RB than in internode 2, whereas in AG there was proportionally less FA present in internode 9 than in internode 2.

Despite the fact that syringyl units and ferulates increased more intensely between internodes in RB, the S/FA ratio was higher in AG for all internodes. The S/PCA ratio could indicate the degree of *p*-coumarate on lignin and, despite the fact that in both genotypes syringyl unit abundance increased with maturity, this parameter remained roughly constant and similar between the genotypes, with the exception being a greater ratio for internode 9 of AG. The FA/PCA ratio is useful in determining which hydroxycinnamate is predominant in the cell wall. The results suggest that in internodes 5 and 9 in both genotypes *p*-coumarate content are greater than that of ferulate (Table 2). However, this observation must be tempered by the fact that PCA is largely found just as a free-phenolic pendant unit, whereas ferulate dimerizes and becomes incorporated into lignins, with both processes depleting the quantitated ferulate and underestimating the total level of ferulates in the wall.

Cellulose deposition was significantly greater in RB compared with all internodes of AG, and in both genotypes cellulose increased with culm development (Table 3). As lignin was lower in the modern hybrid, both changes contribute to higher cellulose/lignin ratio in this genotype. The youngest and intermediate internodes presented the higher cellulose/lignin ratios in both genotypes (Table 3).

**Table 3:** Cellulose, xylan, uronic acid, acetyl groups, cellulose/xylan and cellulose/lignin in RB867515 (RB) and *S*. *spontaneum* (AG) internodes.

Sample	Cellulose	Xylan	Uronic acid	Acetyl groups	Cellulose/Xylan	Cellulose/Lignin
	%				ratio	
RB IN2	53.15 D*	20.80 A*	0.95 A	3.25 A*	2.55 D	3.33 A*
RB IN4	58.60 C*	18.20 B	0.30 B	1.25 B*	3.21 C*	3.32 A*
RB IN5	59.53 B*	17.25 C*	0.30 B	1.15 B*	3.45 B*	3.17 B*
RB IN9	61.90 A*	16.27 D*	0.25 B	0.60 C*	3.78 A*	3.17 B*
AG IN2	51.55 C	20.10 A	1.00 A	4.50 A	2.56 C	2.97 A
AG IN4	55.35 A	18.40 B	0.30 B	2.00 B	3.01 A	3.04 A
AG IN5	53.85 B	18.40 B	0.25 B	1.75 B	2.92 B	2.72 B
AG IN9	53.20 B	17.85 C	0.25 B	1.40 C	2.98 B	2.48 C

Means followed by different letters differ significantly between internodes (Tukey test, p<0.05). Asterisks indicate a significant difference between the genotypes for the same internode (t-test, p<0.05), (n = 3).

Levels of xylan in intermediate and mature internodes were greater in AG whereas a greater content was found in the youngest internode of RB. A significant reduction in xylan deposition in the cell wall was observed during internode maturation (Table 3). The ratio cellulose/xylan was higher in intermediate and mature internodes from RB whereas no significant differences were found for the youngest internode.

Acetyl group deposition decreased significantly with stem maturity and was greater in AG. Uronic acid levels were similar in both genotypes and levels reduced with maturity (Table 3). Most acetate is found on hemicelluloses, but we do not currently know the fraction that is on lignin.

#### The abundance of cell wall ester- and ether-bound phenolics varies throughout culm development

The hydroxycinnamates (ferulate, caffeate, *p-*coumarate) were quantified relatively as their ester and ether forms during culm development (Table 4). Levels of ferulate esters changed during culm development. In RB ferulate ester abundance was greater in IN4 and IN5 relative to IN2 and IN9, whereas in AG the pattern was slightly different with lower levels of ferulate ester in IN9 relative to the other internodes. The pattern of *p-*coumarate ester abundance in the two genotypes was similar, with significant increases throughout culm development in both RB and AG. Caffeate abundance also showed a similar pattern in the two genotypes, with a major reduction in abundance between IN2 and the other three internodes analysed. When comparing the genotypes we detected greater levels of ferulate esters in internode 2 of AG compared to the same internode in RB, whereas the opposite trend was observed when comparing internode 5. Levels of *p-*coumarate esters were greater in IN4 and IN5 of AG compared to RB, whilst levels of caffeate esters were lower in IN2, IN4 and IN5 of AG when compared to the same internodes in RB.

**Table 4:** Abundance of total, esterified and etherified phenols in *S*. *spontaneum* (AG) and RB867515 (RB) through culm development.

Cell wall phenols	RB867515 (RB)				*S*. *spontaneum* (AG)			
	IN2	IN4	IN5	IN9	IN2	IN4	IN5	IN9
**Total phenolic**	1 C	1.24 B	1.30 A	1.31 A	1.08* B	1.28* A	1.30 A	1.36 A
**Ester-bound phenolics**								
Ferulate	1.00 A	1.43 B	1.46 B	1.00 A	1.17* A	1.23 A	1.17* A	1.02 B
*p*-Coumarate	1.00 A	2.64 B	2.74 B	3.56 C	1.34 A	3.16* B	3.78* C	3.78 C
Caffeate	1.00 A	0.07 B	0.02 B	0.01 B	0.48* A	0.02* B	0.01* C	0.01 C
**Ether-bound phenolics**								
Ferulate	1.00 A	4.38 B	4.42 B	3.44 C	1.61 A	3.13 AB	4.77 B	2.73 A
Caffeate	1.00 A	0.31 A	0.31 A	0.21 A	2.89 A	0.04 B	0.54 B	0.45* B

Data are means of relative quantification of total, ester-bound and ether-bound phenolic from CWD material. Values are given relative to the abundance in IN2 of RB. Means followed by different letters differ significantly between internodes (Tukey test, p<0.05). Asterisks indicate a significant difference between genotypes for the same internode (t-test, p<0.05), (n = 3). IN2: internode 2, IN4: internode 4, IN5: internode 5 and IN9: internode 9.

Differences in the ether-bound phenols were also detected throughout culm development. [Etherified phenolics were assumed to also be esterified]. Levels of etherified ferulate, determined by high-temperature base treatment increased from IN2 to IN5 in both genotypes before decreasing in IN9. Etherified caffeate on the other hand showed a decrease between IN2 the more mature internodes, though this difference was only significant for the AG genotype. In general the HPLC-QqQ results agree with trends observed by NMR analysis, although it is important to note the differences in sample preparation and quantification methods between these two techniques. Measurement of total phenolics using the Folin-Ciocalteau method indicated that the levels of these compounds increased with internode maturity in both genotypes, and that their levels were slightly higher in internodes 2 and 4 of AG in comparison with RB.

### Correlation of cell wall composition with saccharification and fermentation

**Fig 5:**
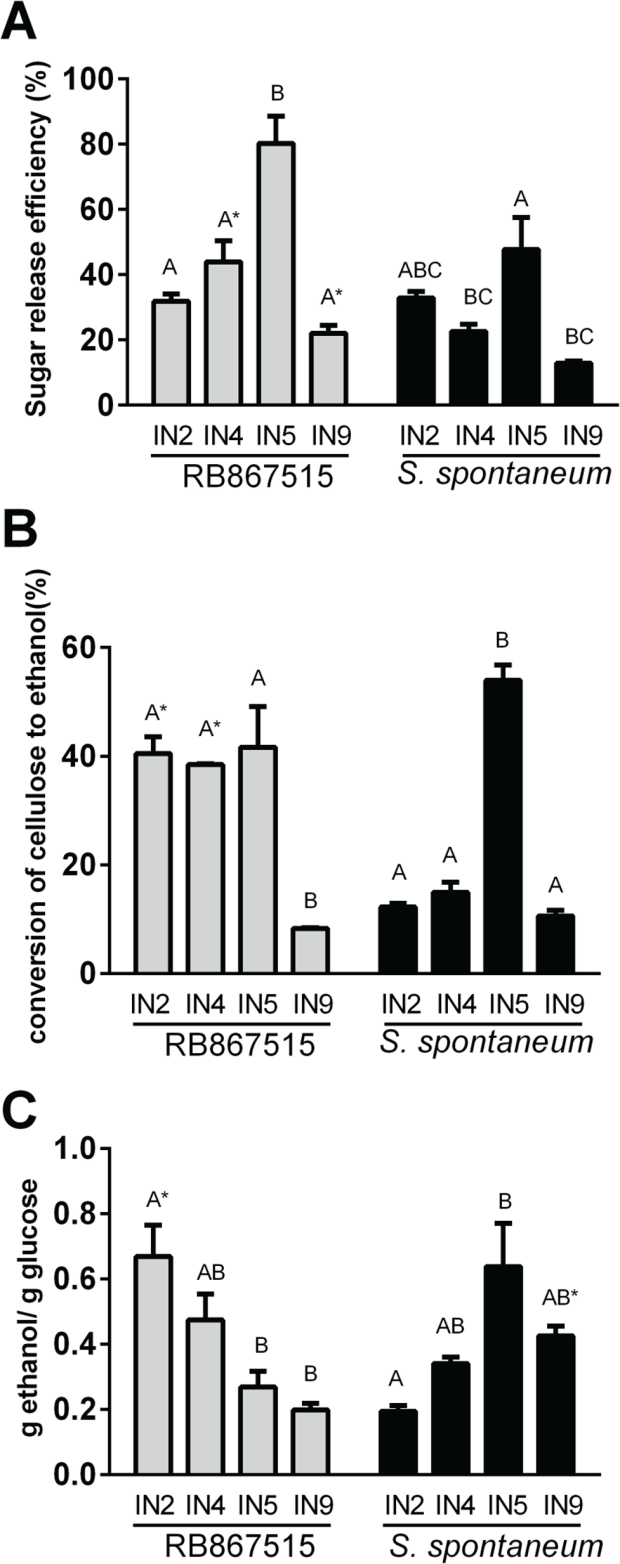
Enzymatic hydrolysis and fermentation. A) Sugar release efficiency (%) in RB867517 and *S*. *spontaneum* in different stages of culm development B) conversion of cellulose to ethanol (%) in RB867517 and *S*. *spontaneum* in different stages of culm development C) g ethanol/ g glucose ratio in RB867517 and *S*. *spontaneum* in different stages of culm development. Values are mean and standard error (n = 3). Black bars–*S*. *spontaneum* (AG), grey bars–RB867515 (RB). Bars with common letters were not significantly different (Tukey test, p<0.05). Asterisks indicate a significant difference (t-test, p<0.05) between the same internodes of the two plants studied. Error bars indicate SE of three biological replicates (n = 3). IN2: internode 2, IN4: internode 4, IN5: internode 5, IN9: internode 9.

Sugarcane bagasse internodes (IN2, IN4, IN5 and IN9) were acid pretreated and subjected to saccharification for 72 h followed by fermentation. The highest sugar release efficiency after 72 h of hydrolysis was obtained for the intermediate internodes (IN5) of RB, reaching 80% and 44% (IN4) (Fig 5A), with lower values for younger and older internodes, suggesting the contribution of different recalcitrance traits.

Cellulosic ethanol production efficiency was significantly greater in the modern hybrid RB but only for young (IN2) and intermediate (IN4) internodes (Fig 5B), despite the fact that higher glucose was released from IN9 for this hybrid (Fig 5B). This difference could be evidence for a greater presence of recalcitrance factors affecting fermentation in the modern cultivar.

The ratio ethanol/glucose is a good indicator of recalcitrance factors that have a specific effect on fermentation (Fig 5C). Whereas in the youngest internode fermentation was more efficient in the modern hybrid, the opposite was found in internode 5 and 9, where fermentation efficiency was higher in AG. This divergence indicates that different recalcitrance factors exist in young and old internodes.

**Fig 6:**
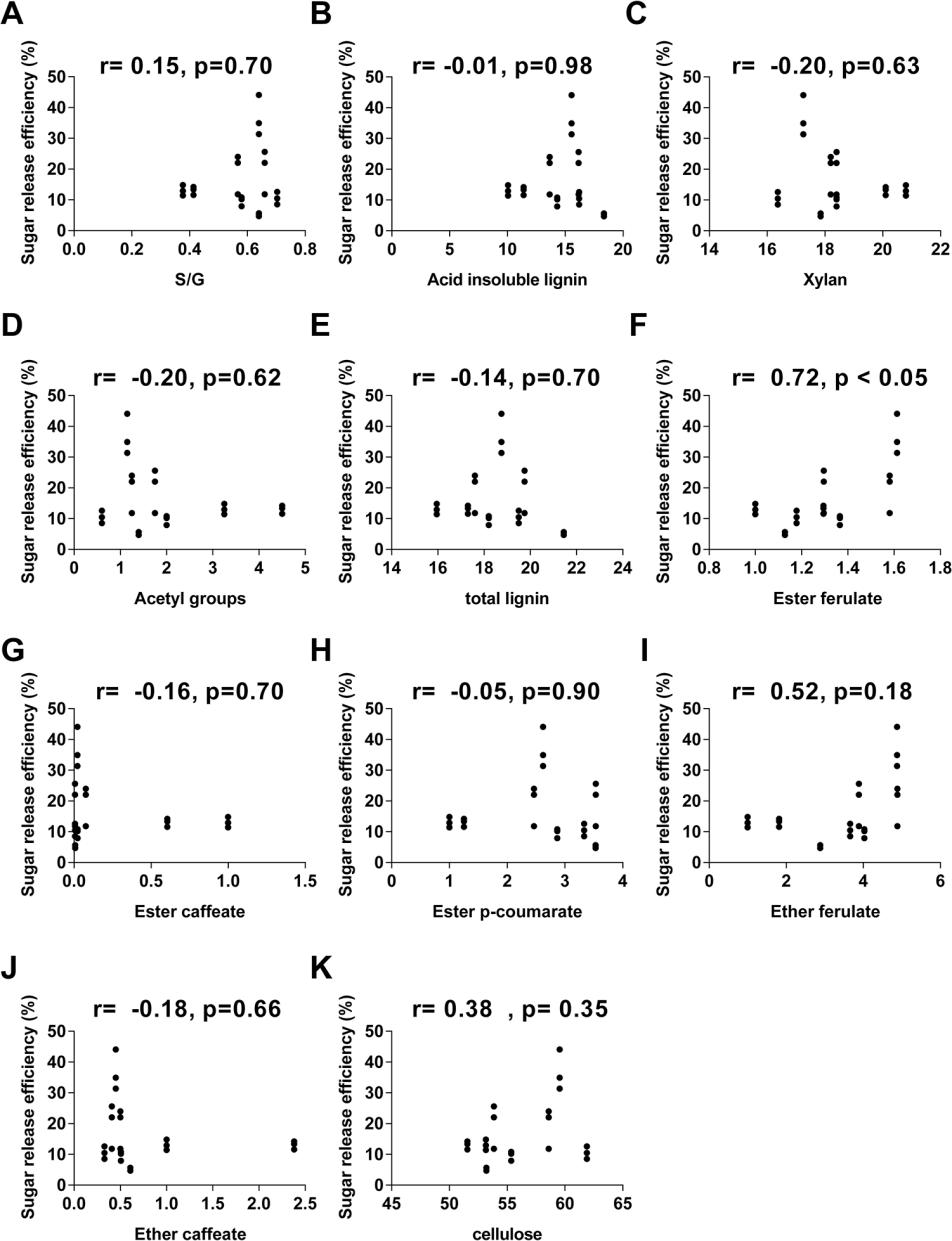
Interactions between cell wall compounds and sugar release efficiency. Scatterplots representing all internodes analyzed of *S*. *spontaneum* (AG) and RB867515 (RB). Sugar release efficiency is shown as a function of (A) S/G ratio, (B) acid insoluble lignin, (C) xylans, (D) acetyl groups, (E) total lignin, (F) ester ferulate, (G) ester caffeate, (H) ester *p*-coumarate, (I) ether ferulate, (J) ether caffeate, (K) cellulose. The Pearson correlation coefficient and its corresponding *p* value are shown at the top of each graph.

**Fig 7:**
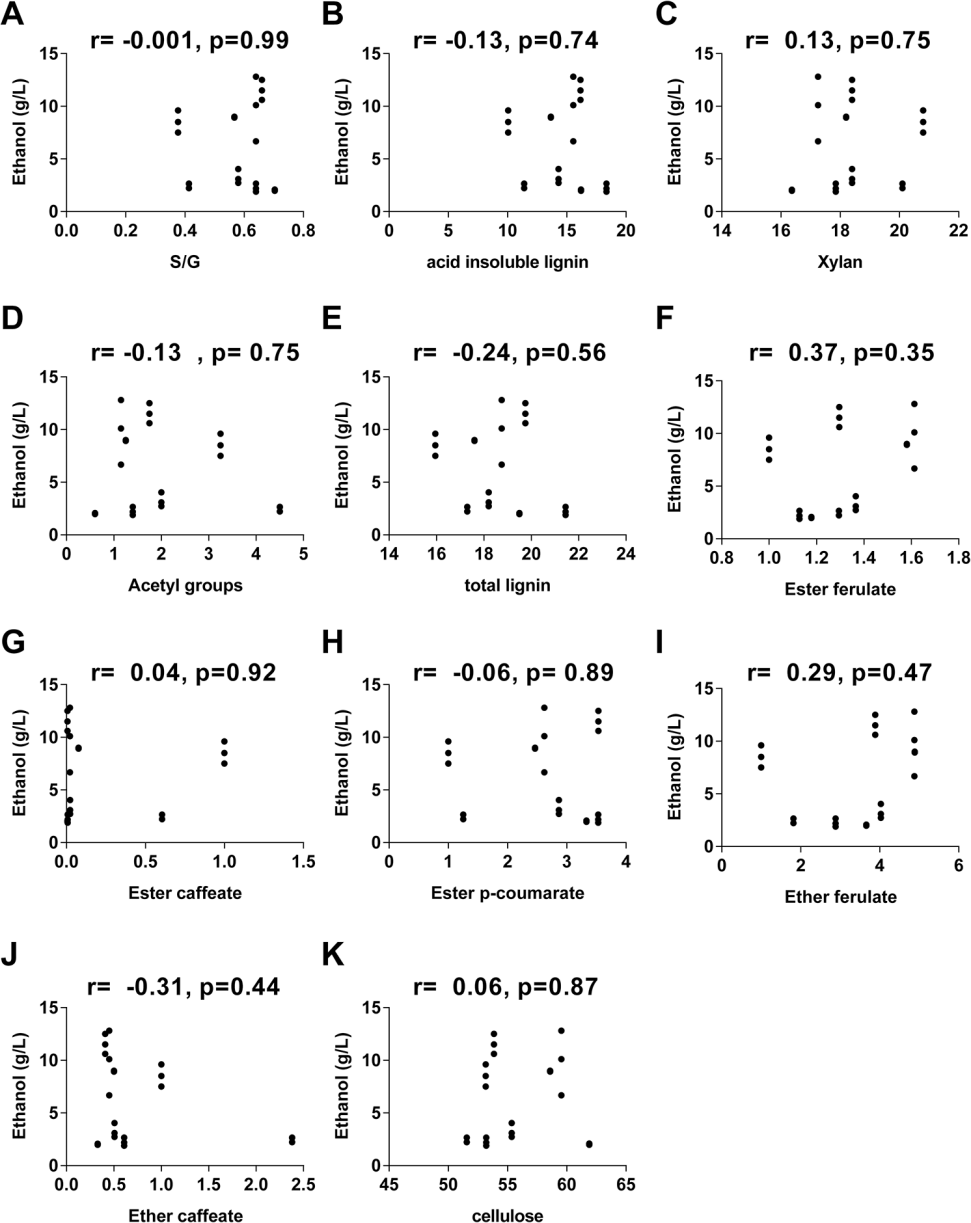
Interactions between cell wall compounds and ethanol production. Scatterplot representing all internodes analyzed of *S*. *spontaneum* (AG) and RB867515 (RB). Ethanol is shown as a function of (A) S/G, (B) acid insoluble lignin, (C) xylans, (D) acetyl groups, (E) total lignin, (F) ester ferulate, (G) ester caffeate, (H) ester *p*-coumarate, (I) ether ferulate, (J) ether caffeate, (K) cellulose. The Pearson correlation coefficient and its corresponding *p* value are shown at the top of each graph.

A correlational analysis was performed to determine the effect of the main cell wall compounds oncellulosic ethanol production. The relationship between cell wall composition and cellulosic ethanol production were determined using Pearson correlation analysis and scatterplot graphs (Figs 6 and 7).

Our results showed a lack of correlation between lignin composition or content and sugar release efficiency (Fig 6A, 6B and 6E). Moreover, among the cell wall components analyzed, the only significant correlation observed was between sugar release efficiency and ester ferulate (*p* value<0.05) (Fig 6F).

Not significant correlations were found between any of the cell wall components analyzed in this study and ethanol production (Fig 7).

## Discussion

### Anatomical and histochemical analyses revealed great differences in culm development between distant genotypes

The anatomical and histochemical analyses showed a greater proportion of lignified cells in the intermediate internode (IN5) as well as significantly higher levels of acid-insoluble lignin content in *S*. *spontaneum* (AG). Curiously, despite the higher amount of fiber cells in the modern hybrid RB, the amount of non-lignified fiber cells was about 2.8 fold higher than in the ancient genotype. In addition, AG also presented longer fiber cells with thicker cell walls, but its metaxylem cells are smaller and thinner. This contrast between genotypes in the anatomical analysis indicates that an increase in fiber cells is not necessarily associated with greater culm lignification. Considering that modern hybrids have taller culms, with less lignified fiber cells (38% less), greater fiber cell number (41.5% more), and thicker metaxylem cells (75% more) compared to AG, these differences suggest that an increase in non-lignified fiber cell number and/or cell wall thickness could compensate for decreased culm lignification. Such a compensatory mechanism was also proposed in the analysis of the *CCR1* maize mutant, where a decrease in lignin content and composition was accompanied by an increase in sclerenchyma surface area [43]. We have carefully analyzed longitudinal sections of sugarcane culm, and the cell features observed allow us to exclude the presence of sclerenchyma tissue in culms of this plant species, classifying it as interfascicular fiber tissue (data not shown).

Both histochemical staining techniques revealed a sharp differential distribution pattern of lignin content and composition in cells and tissues between genotypes during through culm development. These analyses confirmed the anatomical results, showing that fiber cells in *S*. *spontaneum* present higher lignin content whereas the opposite seems to occur in the metaxylem cells. Characteristic Mäule staining of syringyl residues showed that in the ancient genotype there is earlier and greater S lignin deposition in fiber cells, suggesting that different lignin structures exist between genotypes for this cell type. The opposite occurred in metaxylem cells, though this could not be verified by chemical analysis as our methods did not allow for discrimination between tissues. However, other authors using TOF-SIMS imaging have shown that vessel cells are more rich in guaiacyl lignin in poplar [44] and maple [45], whereas syringyl lignin is mainly located in fiber cell walls. On the other hand, laser dissection coupled to pyrolysis GC-MS of several mutants of Arabidopsis have concluded that both xylem and interfascicular fiber cells formed the S-enriched lignin [46]. This contrast illustrates the possibility that tissues with similar function in different plant species could have lignin with different structures. Together, these data suggest that during evolution in sugarcane there occurred an increase in vascular tissue area and xylem cell size as well as a decrease in lignification of fiber tissue. The modern hybrids, with taller and thicker culms, more leaves per culm, and a concomitant reduction in culm number, probably would need an increase in xylem water conductivity and/or xylem area in order to support greater transpiration that occurs with the morphological changes resulting from human selection for higher sucrose production per plant [41].

### Differences in lignin content are greater during culm development than between distant genotypes but are not correlated with saccharification and fermentation yield

Despite significant differences in culm height and diameter, anatomy and histochemical results, and dramatic changes in lignin composition, differences of only 2% in total lignin content were found between the same internodes of the different genotypes. However, within the same culm, the differences in lignin between internodes were around 7% in both genotypes. This contrast suggests that great differences exist in lignin biosynthesis depending on the age of the internode, and that sampling within a single plant could offer a useful resource for the study of recalcitrance or changes in lignin biosynthesis; data obtained from a single plant could be more easily interpreted than comparisons between different genotypes with extensive differences in plant morphology and culm anatomy.

This conclusion is also supported by the known impact of the environment on lignin abundance, as indicated by analysis of field growth of CAD silenced *N*. *attenuata* plants which clearly illustrated how the environment plays an essential role in regulating lignin biosynthesis [47].

A previous study comparing clones of sugarcane hybrids detected variation in total lignin of between 16.8% and 24.5% between 10 different clones, using material from a whole culm bagasse mill [48]. On the other hand, we analyzed these same clones in two other harvests and found a major variation in the amount of lignin, with the clones accumulating the greatest amount of lignin in one harvest being different from the clones accumulating higher lignin levels in a following harvest (data not shown). This strong influence of environment on lignin between genotypes suggests that variation in lignin within the same plant could be a more useful strategy in the study of variation in lignin metabolism. In this study despite differences in the temporal profile of lignin deposition between the ancient and the modern hybrid, there are considerable similarities in the spatial pattern of lignin deposition between these two genotypes.

Despite the changes we detected in lignin content, we did not find a correlation between lignin content and either saccharification or fermentation efficiency. Numerous studies indicate a negative impact of lignin on enzymatic hydrolysis. The majority of studies that present good evidence for a negative effect of lignin on enzymatic hydrolysis or digestibility were carried out using transgenic plants or with mutants under greenhouse conditions [8,49,50]. Changes were always compared with WT plants, and most of these studies did not include analysis of other metabolites that also affect recalcitrance, for example other phenols, lignans, or their derivatives. Such compounds may also be affected in plants with altered lignin abundance, for example transgenic tomatoes with reductions in lignin content exhibited a dramatic increase in pools of soluble phenols [51]. In several Arabidopsis mutants with decreases in monolignol biosynthetic enzymes, it has been demonstrated that a number of classes of phenylpropanoids accumulate, and other complex perturbations in primary and secondary metabolism were also revealed both by transcriptomic and metabolomic analyses [52]. Studies also exist that explore the effects of natural variation, some of which indicate lignin as major recalcitrance factor and some of which do not indicate lignin as being of great importance. Studer et al. [12] analyzed 1,100 wild accessions of *P*. *trichocarpa* and found no significant correlations between lignin and enzymatic hydrolysis efficiency. These authors have postulated that others factors beyond lignin affect recalcitrance. Penning et al [53], analyzed a large inbred population over several years and have mapped 5 QTLs for lignin and 4 for enzymatic hydrolysis yield; these QTLs do not share the same genes, indicating the polygenic nature of recalcitrance in maize. More recently, the screening of a mutant population of *Brachypodium distachyon* showed lines with high saccharification efficiency associated with alterations in cell wall compounds other than lignin [54].

Studies using isolated lignins from different species have shown that lignin from some species inhibits cellulose hydrolysis, whereas that extracted from other species does not [55]. Other lines of evidence also support a minor role of lignin in recalcitrance [56].

NMR measurements indicated generally lower percentage abundance of guaiacyl and greater percentage abundance of syringyl lignin units in RB, explaining the higher S/G ratio in this genotype. The S/G ratio determined for RB IN9 using NMR (1.3) was similar to that reported for corn stalks using the same technique (1.41) [57]. Chemical analysis also revealed a significantly higher S/G ratio in internode 9 of RB, and both methods indicated increasing S/G ratio with internode maturity in both genotypes. However, we did not uncover a significant correlation between S/G ratio (chemical analysis) and enzymatic hydrolysis yield (*p* value = 0.70) or fermentation efficiency (*p* value = 0.99). Although several studies have reported higher S/G as a negative factor in saccharification [58–60], analysis of several genotypes of wheat and rice suggested that the S/G was not the major factor affecting enzymatic hydrolysis [61]. The same was proposed in an analysis of 1,100 undomesticated *P*. *trichocarpa* accessions [12]. Using a model system, it has been shown that lignin composition *per se* has less effect on cell wall fermentation kinetics [62,63].

### The majority of differences in cell wall composition are not correlated with saccharification efficiency

High levels of cellulose in the cell wall are an important factor for the production of more cellulosic ethanol from biomass. The modern hybrid RB867515 (RB) contains more cellulose in all internodes, by from 3% to 16% of mass, with the difference increasing progressively with internode maturity. However this progressive increase in cellulose is not correlated with saccharification efficiency (*p* value = 0.35), a fact that was clearly demonstrated by the sharp reduction in cellulose conversion to glucose that occurs between internodes IN5 and IN9. Analysis of several Arabidopsis mutants for lignin biosynthetic enzymes [64] also did not reveal correlations between cellulose content and enzymatic hydrolysis efficiency. These results suggest that positive effects of higher cellulose content could be completely overridden by natural variation in recalcitrance factors.

Plant cell walls contain acetyl groups which, between the groups of cell wall polysaccharides, mainly belong to arabinoxylan, but are also found on xyloglucans, homogalacturonans and rhamnogalacturonans [65]. Acetylation can also occur on lignin, as detected in grasses [66–68]. In both cases, acetylation is postulated to potentially reduce cellulosic ethanol production, affecting both saccharification and/or fermentation [65,69,70]. However, total cell wall acetylation, despite being higher in *S*. *spontaneum* for all internodes analyzed (between 1.4 and 2.3 times the values in RB), decreased progressively with maturity in both genotypes and was not correlated with saccharification (*p* value = 0.62). It is possible that the variation in recalcitrance traits between these genotypes is so high and unpredictable that it overcomes the significance of individual correlations with the efficiencies of hydrolysis or fermentation. This could also explain the lack of correlation between lignin content or S/G ratio and efficiencies of hydrolysis or fermentation. This hypothesis is in agreement with the growing evidence that recalcitrance to enzymatic hydrolysis of biomass is governed by quantitative inheritance that is polygenic in nature, and strongly affected by the environment [53]. The complex nature of this recalcitrance is also supported by studies of biomass biodigestibility [71,72]. Recent studies have also shown that biodigestibility recalcitrance is closely related to saccharification recalcitrance [73,74]. Biodigestibility studies are also potentially important guides for decreasing recalcitrance to cellulosic ethanol production, as traditional breeding of forages has focused on increasing total dry matter digestibility rather than cell wall digestibility, which has in turn resulted in minimal reductions in cell wall lignification, and as a consequence, fewer deleterious effects on plant growth and defense [75,76].

Although NMR indicated that *p*-coumarate (PCA) and ferulate (FA) increased with stem maturity in relation to lignin, there was no apparent relationship with saccharification yield. The dramatic decrease in enzymatic hydrolysis efficiency between IN5 and IN9 was associated with opposing changes in PCA between the genotypes; PCA decreased in the ancient genotype whilst increasing the modern cultivar. In addition, no significant correlations were found between ester or ether-linked PCA or ferulate abundance determined using UPLC-QqQ MS and saccharification yield

We found an increase in ester-bound PCA with stem maturation as previously reported for other plant species [77]. Again it is important to exercise caution due to the limitations in the methods for determining ester/ether-linked hydroxycinnamates. Extensively used in digestibility studies, and rarely in lignin chemistry, these forms of these acids were more negatively correlated with digestibility than with lignin [78]. However, the dramatic reduction in saccharification in internode IN9 was associated, in general, in both genotypes, with a decrease in etherified FA, explaining the low correlation between the content of this component and enzymatic hydrolysis.

We found only one significant correlation, between esterified FA and enzymatic hydrolysis (*p* value = 0.02). A high correlation between hydrolysis yield and esterified FA was also found in switchgrass (*p* value <0.0001) [79] but was not found for maize [27]. The interpretation of the effect of esterified FA on saccharification remains contentious. Monolignol-ferulate conjugates, as opposed to monolignol-*p*-coumarates, are compatible with normal lignification reactions and can be integrated into the lignin polymer [25], but are currently unknown (or at very low levels) except in transgenic plants incorporating exotic transferase genes [80]. This conjugate has an easily cleavable ester bond that, when introduced into lignin, increases saccharification yield following mild base pretreatments [80]. Although these conjugates are considered exotic, grass lignins have very high amounts of ferulate with ester ligations, a proportion of which could be linked to monolignols, resulting in more labile linkages being incorporated into lignin, which in turn could explain the positive correlation between ester-bound ferulate and saccharification efficiency.

Although NMR analysis indicates that *p*-coumarate content is greater than ferulate in sugarcane, and PCA acylates both monolignols and hemicelluloses, we did not find correlations between ester-bound coumarate and enzymatic hydrolysis. This result contrasts with that of Zhang et al. (2011) who found in maize that esterified *p*-coumarate content showed a significant and high negative correlation with *in vitro* degradability, and with Taboada et al. (2010) where correlation between digestibility and esterified FA was not observed in several silages. Even though it is assumed that most of the ferulate in grass cell walls is on arabinoxylan polysaccharides, ferulates are known to be involved in lignification and, in addition to being etherified to lignin in grasses [81–84], they are also involved in many other structures [25]. As commented by Wilkerson et al. (2014) it is difficult to detect ferulate in the lignin polymer which makes accurate quantification or detailed studies about the natural variation in this incorporation almost impossible with current technology. There is one more possibility that is being explored in the Ralph labs. The recent modification of the DFRC method coupled with GC-MS triple-quadrupole detection is now capable of detecting monolignol ferulate conjugates that have been incorporated into lignin [80]. Although the low content and the extremely low release of these nevertheless diagnostic conjugates hinders their analysis, an assessment of the native levels in bioenergy crops is underway. Altogether, the criticism and controversies clearly illustrate how much we need to learn about lignin structure and, more importantly, how lignin structure affects lignin functions and degradability.

### Variation in culm development has profound effects on enzymatic hydrolysis and fermentation efficiency

The higher cellulose conversion efficiency of internodes IN5 in both genotypes contrasts with the decrease in hydrolysis yield with internode maturity that was also described in switchgrass [79]. The analysis of ethanol production normalized for the glucose produced during fermentation reveals that the ancient genotype permits a higher efficiency of ethanol production in IN5 and IN9, indicating that the modern hybrid has higher fermentation recalcitrance, despite its high enzymatic hydrolysis. These contrasting genotypes give support to the idea that different recalcitrance factors could affect saccharification, fermentation, or both, and that breeding programs in sugarcane can result in increases in some of these recalcitrance factors. However, in this study, it was not possible to reveal the identity of these recalcitrance factors using correlative analysis, and it appears that recalcitrance is probably due to combined effects of overall anatomical, morphological and chemical composition of the cell walls. Despite the elusive nature of these recalcitrance factors, our data clearly indicates that factors beyond lignin that affect recalcitrance exist. Another often unaddressed issue is the difficulty of finding analyses that fully identify the role of a component; for example, ferulates and diferulates that are involved in lignification and become bound up in the polymer can only partially be released [25,85], and it may never be possible to actually quantify the true level of involvement. On the other hand, the small differences in cell wall composition that we detected, despite clear differences in recalcitrance, suggest that the analysis of variation in cell wall composition and its effects on recalcitrance could be more informative, and less subject to environmental effects than comparisons between genotypes with differences in cell wall composition.

## Materials and Methods

### Plant materials and growth conditions

Plants of genotypes RB867515 (RB) and *S*. *spontaneum* (AG) were grown in 15 kg pots containing a 1:1 mixture of Tropextrato potting mix (Plantimax) and maintained in a growth chamber conditions at Universidade Federal de Viçosa (UFV). Culms were harvested from one year old plants and only the region between nodes (internodes) was collected. Internodes were numbered counting from the stem apex: the youngest internode (IN2), the intermediate internodes (IN4 and IN5) and the mature internode (IN9).

The experimental design was completely randomized in a 2 x 4 factorial arrangement, with the first factor corresponding to the genotypes and the second to the internodes with three replicates and one plant per plot. For micromorphometric analyses the factorial arrangement was 2 x 1 with five replicates. The first factor corresponding to genotype and the second to the intermediate internode (IN5).

### Micromorphometric analysis

The intermediate internode IN5 was collected and fixed in formalin-acetic acid- 50% alcohol, 1:1:18 (FAA)_50_ for 48 h [86] and preserved in 70% ethanol. Samples were sectioned in a sliding microtome (Leica) (120 μm thick cross sections), stained with safrablau (safranin 1% m v^-1^ and astra blau 0.1% m v^-1^ at the rate of 7.5:2.5) [87] and mounted in glycerinated gelatin. Samples from the same tissue were softened in ethylenediamine for 10 days and embedded in methacrylate (Historesin, Leica Instruments, Heidelberg, Germany). Cross and longitudinal sections of 5 μm were obtained with an automatic advance rotary microtome (model RM2155, Leica Microsystems, Deerfield, Illinois, USA), stained with toluidine blue pH 4.0 [88] followed by Lugol’s iodine solution. The fixed sections were mounted on slides using synthetic resin (Permount).

To obtain individual cells, a maceration method with hydrogen peroxide solution (30% v/v) and acetic acid 1:1 for 48 h at 60°C and staining with safrablau was used. Images were obtained with a light microscope (model AX70TRF, Olympus Optical) and analyzed with software Image Pro-Plus 4.5 (Media Cybernetics, InC., USA).

The following anatomic characteristics were measured: total cross-sectional area of the stem, vascular tissue area, lignified fibers area, not lignified fibers area, total fiber area (lignified + non lignified), fiber length, fiber with, cell wall thickness and lumen diameter, diameter of parenchyma cells, diameter and thickness of metaxylem vessels. Parenchyma cells and metaxylem vessels were measured within the pith region. Data was processed as a percentage relative to the total cross-sectional area.

### Cell wall histochemical analysis

Stem material from internodes two (IN2), five (IN5) and nine (IN9) counting from the stem apex were fixed in FAA_50_ for 48 h [86] and stored with 70% ethanol. Transverse sections from IN2 were obtained in a table-microtome (LPC Rolemberg & Bhering) and IN5 and IN9 with a sliding microtome (Leica SM2000R). Lignin deposition and composition were determined using Wiesner reagent [86] and Mäule reagent [89]. Images were captured using an optical microscope (Olympus Optical model AX70 TRF) with a digital camera (Spot Insightcolour 3.2.0, Diagnostic Instruments Inc.) and analyzed with the images Spot Basic software. The Mäule reaction was detected with polarized light.

### Plant cell wall extraction

Internodes: two (IN 2), four (IN4), five (IN5) and nine (IN9) from RB and AG were dried at 75°C until constant weight. Dried material was homogenized to a fine powder using a Wiley mill (Thomas Scientific). Particle size was selected with a 40–60 mm mesh screen. Homogenized samples were treated with acetone for 5 h in a Soxhlet apparatus. The extracted cell wall material (CWM) was used for further analyses.

### Chemical analysis of cell wall

Chemical characterization of sugarcane bagasse was performed using the following procedures: acid soluble lignin [90], Klason lignin [91], lignin S/G ratio [92], preparation of biomass for sugar analysis [93], sugar analysis [94], acetyl groups [95] and uronic acids [96].

### Nuclear magnetic resonance (NMR) spectra

Approximately 50 mg of ball-milled cell walls were used to obtain NMR spectra. Sample preparation, spectroscopy and spectral analysis was carried out as described in [57].

### Analysis of phenolic compounds

Phenolic content was measured with a method based on the reduction of phosphomolybdate/phosphotungstate with Folin-Ciocalteau reagent (Sigma). Samples (25 mg of CWR) were treated with 2 N NaOH for 18 h at room temperature. After alkaline hydrolysis, the solution was adjusted to pH 2.0 with HCl and immediately resuspended in methanol 70%. After several washes, a Speedvac was used to concentrate the sample and resuspend in methanol 70% (200μL). Folin-Ciocalteau reagent (1:10) and a sodium carbonate solution 7.5% were added to the extraction.

Samples were homogenized and incubated at room temperature for 30 min followed by determination of phenolic compounds using a microplate reader (ELISE Versamax Molecular Devices, Sunnyvale, CA), at 765 nm. Gallic acid was used for the standard curve.

### Analysis of ester- and ether-bound cell wall phenolics

Ether and ester-bound cell wall phenolics (ferulate, *p*-coumarate, caffeate) and released vanillin were extracted and analyzed according to [97] with some modifications. CWM (25 mg) ester-bound cell wall phenolics were analyzed using the total phenolic extract in 4 N NaOH for 18 h at room temperature; the ether-bound cell wall phenolics were released in 4 N NaOH for 2 h at 170°C under a nitrogen atmosphere. After alkaline hydrolysis, the samples were acidified with HCl to pH 2.0. The phenolics were extracted three times with ethyl acetate. Then, the samples were evaporated under vacuum and resuspended in 70% methanol. The analysis of the resulting free acids was performed using a UPLC-QqQ MS (Triple Quadrupole 6430, Agilent Technologies, Waldbronn, Germany) equipped with an Eclipse Plus C18 (2.1 x 50 mm, 1.8 μm) column and guard column at a flowrate of 0.2 ml/min. The mobile phase was formic acid 0.1% (Solution A) and 0.1% formic acid in acetonitrile (solution B), and the gradient program was: 4 min 90% solution B, 4.5 min solution B, 6 min 19% solution B. For the Triple-quadrupole mass spectrometer the instrument parameters were: negative-ion mode, capillary voltage 4.0 kV, flow rate of desolvatation gas 13 L.min^-1^; nebulizer gas 35 psi, source temperature 340°C, cone voltage 135 V, dwell time 100 s, fragmentor 88 eV, collision energy and transitions: 12 eV (ferulic acid 193 > 134), 13 eV *p*-coumaric acid 163 > 119 and caffeic acid 179 > 119) and 20 eV (vanillin 151 > 108).

### Saccharification and fermentation

Tissue samples from internodes (IN2, IN4, IN5 and IN9) were dried and homogenized to a fine powder using a Wiley mill (Thomas Scientific) before acid pretreatment. Samples at a concentration of 10% (w/v) in 0.5% (v/v) H_2_SO_4_ were pretreated by autoclaving at 121°C for 30 min. The solid fraction was separated by vacuum filtration, washed with 500 mL distilled water and dried at 50°C until constant weight.

Saccharification was performed using the solid fraction obtained after pretreatment of the substrate at 8% (w/v) in duplicates at 50°C and 180 rpm. The solid fraction was suspended in fermentation medium (yeast extract, 2.5 g/L; peptone, 2.5 g/L; NH_4_Cl, 2 g/L; KH_2_PO_4_,1 g/L and MgSO_4_.7H_2_O, 0.3 g/L) in citrate buffer (50 mM, pH 4.8), supplemented with the commercial cellulase (Celluclast, Sigma-Aldrich, Brazil) at the concentration of 15 Filter Paper Units (FPU) per gram of substrate.

After 72 h of saccharification, fermentation was initiated by inoculating with *S*. *cerevisiae* LBM1 cultures to an OD_(600nm)_ of 2 under sterile conditions and conducted at 37°C. Samples were collected after 8 h and centrifuged for 15 min at 10,000 g. The supernatants were used to quantify glucose and ethanol. For ethanol quantification, reaction buffer contained Tris-Base 0.6 M pH 9.7 glycine 0.4 M, NAD^+^ 5 mM. The reaction was stabilized for 5 min at 340 nm before adding alcohol dehydrogenase (Sigma-Aldrich) 1 U/reaction. Glucose was quantified using an enzymatic reaction at 340 nm using a microplate reader (ELISA Versamax, Molecular Devices, Sunnyvale, California, USA). Reaction buffer containing imidazole 100 mM (pH 6,9); MgCl_2_ 5 mM; NAD^+^ 2 mM; ATP 1 mM, glucose 6-phosphate dehydrogenase (2 U/L). The reaction was stabilized for 5 min at 340 nm before adding hexokinase (Sigma-Aldrich) 0.2 U/reaction.

Calculations for sugar release efficiency were performed as described in [98] conversion of cellulose to ethanol (%) were performed as described in [99].

### Statistical analysis

Experiments were performed with three biological replicates or a pool of biological samples for the lignin analysis. Data are expressed as mean±SE. The data were analyzed by ANOVA followed by Student’s t tests or Tukey’s test as a *post hoc* comparison All statistical analyses were carried out with the software Statistic 8.0 using a 95% confidence interval (α = 0.05). Determination of Pearson correlation coefficients was performed among cell wall compounds and sugar release or ethanol production using data from all internodes and genotypes. Coefficients were considered significant for *p* values <0.05.

## Supporting Information

S1 Table Lignin content, composition and S/G lignin ratio of RB867515 and *S*. *spontaneum* internodes. (DOCX)
