# Supplementary material for: Analysis of a Modern Hybrid and an Ancient Sugarcane Implicates a Complex Interplay of Factors in Affecting Recalcitrance to Cellulosic Ethanol Production
Source: PLoS One. 2015 Aug 7;10(8):e0134964. doi: 10.1371/journal.pone.0134964 (PMC4529190; doi:10.1371/journal.pone.0134964)
Supplement: S1 Table — (DOCX) [file pone.0134964.s001.docx]

**Table S1: Lignin content, composition and S/G lignin ratio of RB867515 and *S. spontaneum* internodes**. Means followed by common letters were not significantly different (Tukey test, P<0.05). Asterisks indicate a significant difference (t-test, P<0.05) between same internodes of plant materials studied (n=3). H: hydroxyphenyl, G: guaiacyl, S:syringyl, IN2: internode 2, IN4: internode 4, IN5: internode 5 and IN9: internode 9.

| **Sample** | **Klason lignin** | **Acid soluble lignin** | **Total lignin** | **H** | **G** | **S** | **S/G** |
| --- | --- | --- | --- | --- | --- | --- | --- |
|  | ---------------------------------------------------------%-------------------------------------------------------- | | | | | | |
| RB867515 IN2 | 10.05 D* | 5.90 A | 15.95 D* | 1.00 A | 2.63 C* | 1.00 | 0.38 C* |
| RB867515 IN4 | 13.65 C* | 3.95 B | 17.60 C* | 1.00 A | 3.00 B | 1.70 | 0.56 B |
| RB867515 IN5 | 15.55 B* | 3.20 C* | 18.75 B* | 1.00 A | 3.10 AB | 2.00 | 0.64 A |
| RB867515 IN9 | 16.20 A* | 3.35 C* | 19.50 A* | 1.00 A | 3.23 A | 2.26* | 0.70 A* |
|  |  |  |  |  |  |  |  |
| *S. spontaneum* IN2 | 11.40 D | 5.90 A | 17.30 D | 1.00 A | 2.43 C | 1.00 | 0.41 C |
| *S. spontaneum* IN4 | 14.30 C | 3.90 B | 18.20 C | 1.00 A | 3.00 B | 1.73 | 0.57 B |
| *S. spontaneum* IN5 | 16.15 B | 3.60 C | 19.75 B | 1.00 A | 3.10 AB | 2.03 | 0.65 A |
| *S. spontaneum* IN9 | 18.35 A | 3.10 D | 21.45 A | 1.00 A | 3.20 A | 2.03 | 0.63 A |
